# An invasive prey alters local and landscape contributions of sources and sinks for an endangered predator

**DOI:** 10.1002/ecy.70242

**Published:** 2025-11-16

**Authors:** Meghan A. Beatty, Ismael V. Brack, Robert D. Holt, Denis Valle, Robert J. Fletcher

**Affiliations:** ^1^ Department of Wildlife Ecology and Conservation University of Florida Gainesville Florida USA; ^2^ School of Forest, Fisheries and Geomatics Sciences University of Florida Gainesville Florida USA; ^3^ Department of Biology University of Florida Gainesville Florida USA; ^4^ Department of Zoology, Conservation Research Institute University of Cambridge Cambridge UK; ^5^ Present address: K. Lisa Yang Center for Conservation Bioacoustics Cornell Lab of Ornithology Cornell University Ithaca New York USA

**Keywords:** biological invasion, dispersal, emigration, invasive species impacts, long‐term studies, metapopulation, population dynamics, predator–prey dynamics, source‐sink dynamics, time since invasion

## Abstract

Source‐sink dynamics are a cornerstone of theory for spatially structured populations. Despite long‐standing interest, understanding temporal variation in source‐sink dynamics in wild populations remains rare. Biological invasions have the potential to alter source‐sink dynamics for native species, which may change over time as invasions proceed. We used 28 years of data on reproduction, movement, and survival to estimate annual source‐sink dynamics across the entire range of the endangered Everglade snail kite (*Rostrhamus sociabilis plumbeus*) during the invasion of a novel prey species, the island apple snail (*Pomacea maculata*). Snail kite populations underwent striking changes in source‐sink dynamics with time since invasion, and no population was consistently a source or sink over time. Some initial benefits of increased prey availability on snail kite demography were diminished in the long term. Populations invaded by *P. maculata* impacted uninvaded populations via changes in snail kite retention (i.e., lack of movement) and emigration across the metapopulation. Our findings illustrate how effects of biological invasions can change over time and may take decades to fully emerge, and they emphasize how an invasive species can have distant impacts on uninvaded populations via fluctuations in native species' local retention and emigration. In addition, our results demonstrate how fluctuating emigration and retention alter long‐term interpretations of source‐sink dynamics through variation in local versus landscape contributions of populations to the metapopulation, highlighting that the status of “source” or “sink” can be highly variable through time.

## INTRODUCTION

Understanding how local populations contribute individuals to regional population (metapopulation) growth has long been of ecological, evolutionary, and conservation interest (Holt, 1985; Levin, 1976). Metapopulation theory explores how the spatial structure of populations and movements among them can contribute to metapopulation persistence and spatially varying abundance (Hanski & Simberloff, 1997). We use the term “metapopulation” to denote any spatially structured population where discrete local populations are connected by dispersal (Hanski, 1998). Metapopulations can fluctuate over time in dynamic environments (Hanski, 1999a), such as those with temporally varying connectivity (Perry & Lee, 2019) or habitat quality (Gonzalez & Holt, 2002). To understand metapopulation dynamics, two general approaches have been applied (Fletcher & Fortin, 2018; Hoopes & Harrison, 1998; Ims & Yoccoz, 1997). First, both theory and empirical efforts have focused on colonization‐extinction dynamics to understand the persistence of spatially structured populations (Hanski, 1999b; Risk et al., 2011; Sutherland et al., 2014); such approaches are particularly useful when limited demographic data are available. Second, site‐specific demography has been used to help understand the interplay of local and regional population processes responding to environmental change. Such approaches can provide rich insights into population dynamics, including source‐sink dynamics (Liu et al., 2011; Pulliam, 1988).

**FIGURE 1:**
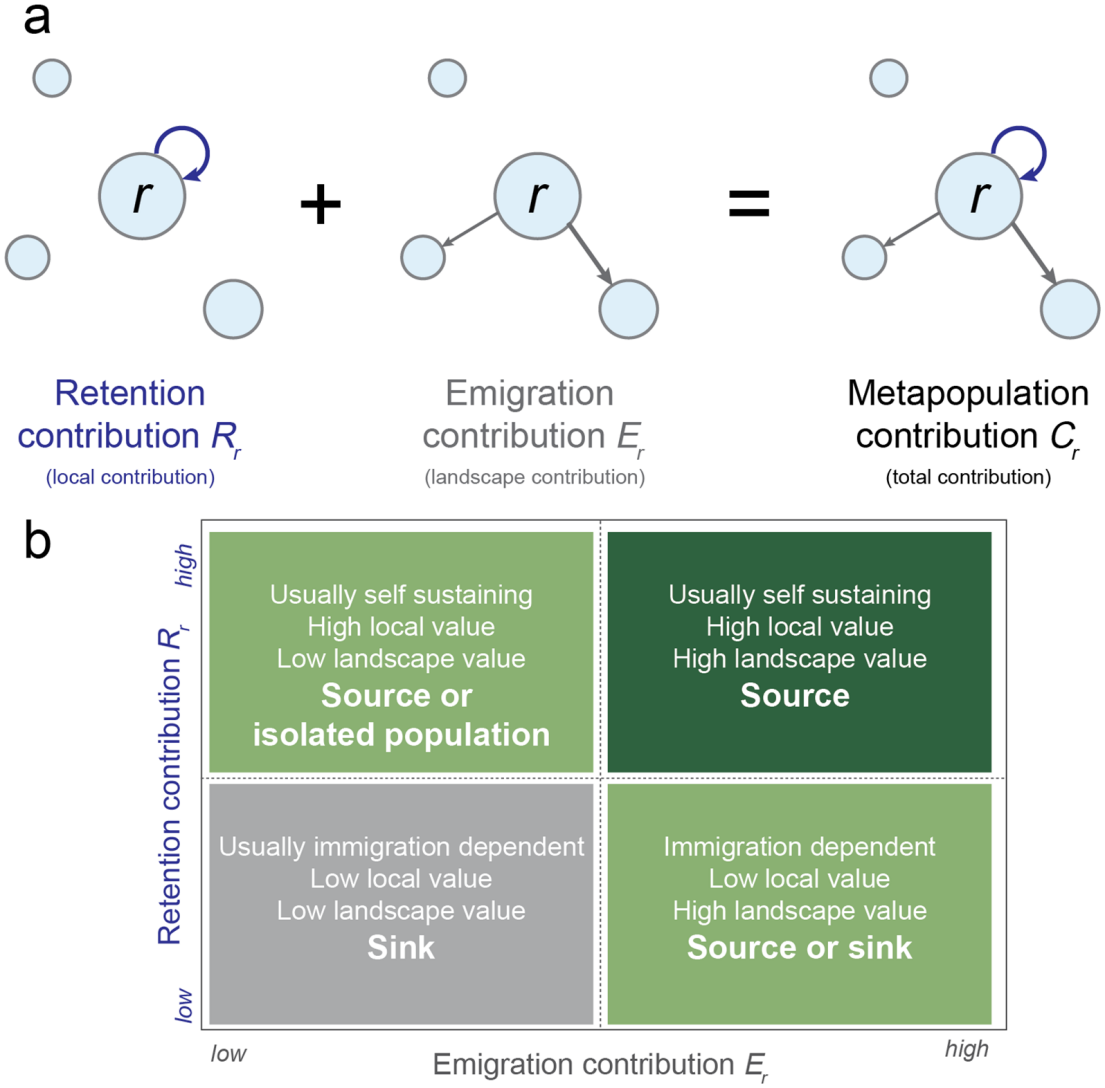
(a) The relationship between $C_{r}$ (the per capita contribution of a member of a local population *r* to the metapopulation), $R_{r}$ (self‐recruitment and retention), and $E_{r}$ (emigration; sensu Runge et al., 2006). (b) The relationship between varying rates of emigration and retention in respect to local and landscape‐scale value to the metapopulation and source‐sink status.

While definitions have varied over time, in general, source populations are deemed to be those that are net exporters of individuals, where births exceed mortality, whereas sinks are net importers of individuals and mortality exceeds births (Loreau et al., 2013; Pulliam, 1988). Here, we used the definition and associated contribution metric ($C_{r}$) proposed by Runge et al. (2006, see also Hixon et al., 2002) that explicitly incorporates emigration into source‐sink estimates to quantify the contribution of a local population to the entire metapopulation. Individuals in a local population can contribute to the metapopulation in two ways: (1) through local birth or survival without emigration (i.e., retention) and (2) through emigration to other populations (Pulliam, 1988; Thomas & Kunin, 1999) (Figure 1). Knowledge of whether source‐sink status is driven largely by local or landscape dynamics can help identify determinants of metapopulation persistence (Sanderlin et al., 2011). Such understanding can support conservation objectives, including informing whether to restore habitat locally or improve connectivity between populations (Akçakaya et al., 2007; Liu et al., 2011; Sample et al., 2020).

Long‐term studies can provide important insights into source‐sink dynamics that would be otherwise obscured. For example, in a 35‐year study of a single population, Badia‐Boher et al. (2025) estimated a long‐term shift from sink to stable status, with an important contribution from floaters (i.e., nonbreeding individuals). The temporal and spatial scale of source‐sink assessments has increased over time, but most studies are relatively short (<10 years) and occur at local or regional scales (Heinrichs, Walker, et al., 2019). Our understanding of long‐term temporal variation in source‐sink dynamics across geographic ranges is therefore limited.

Temporal variation in source‐sink dynamics could be driven by changes in habitat quality (Heinrichs, Lawler, et al., 2019), such as those arising from biological invasions. Invasive species can alter habitat and impact the demography of native species (Cameron et al., 2016; Crystal‐Ornelas & Lockwood, 2020; Pyšek et al., 2012) and could therefore be used to inform our understanding of metapopulation dynamics. Invasive species at higher trophic levels can drive declines (at times devastating) of native species at lower trophic levels (Bradley et al., 2019; Thomsen et al., 2014). In contrast, invasive species at lower trophic levels may benefit higher trophic levels by boosting food supply, leading to increased native consumer abundance (Pintor & Byers, 2015). Notably, the overall impacts of invasion are expected to change with time since invasion (Iacarella et al., 2015; Strayer et al., 2006).

Three temporal patterns are commonly observed or assumed for changes in invasive species abundance following invasion (Appendix S1: Figure S1). First, invasive species may rapidly increase in abundance with time since invasion. Second, after this initial increase in abundance, population growth may slow over time as invasive populations experience density dependence; and third, the invasive population may exhibit boom‐bust dynamics (Lockwood et al., 2013; Strayer et al., 2017). Similar temporal patterns may also describe shifting impacts of the invader on native species. Despite evidence for invasive species influencing the demography of native species, how invasions alter source‐sink dynamics of native metapopulations remains poorly understood (but see Lejeune et al., 2023; Woodford & McIntosh, 2010).

The Everglade snail kite (*Rostrhamus sociabilis plumbeus*; snail kite hereafter), an endangered wetland‐dependent raptor in the United States, presents a unique opportunity to investigate long‐term population dynamics at a broad spatial scale. Here, we used 28 years of individual demographic data to examine temporal variation in source‐sink dynamics across the entire metapopulation of the snail kite during the invasion of a novel prey species—the non‐native island apple snail (*Pomacea maculata*). This snail provided a supplementary food source for snail kites well beyond the supply of native snails (Wilcox & Fletcher, 2016). This increased prey availability initially led to increases in reproduction, survival, body mass, and population growth of the snail kite (Cattau et al., 2016, 2018; Fletcher et al., 2024; Poli et al., 2022). However, it remains unclear if and how source‐sink dynamics of snail kite populations changed because of the *P. maculata* invasion.

Our objective was to examine how snail kite vital rates and emergent source‐sink dynamics changed with time since the invasion of this novel prey. The *P. maculata* invasion resulted in short‐term increases in snail kite reproductive output in the years immediately following establishment (Cattau et al., 2016), but long‐term effects remain unknown. Predator and prey population dynamics should be coupled, particularly for specialized predators such as the snail kite. We predicted invasion by *P. maculata*: (1) temporarily increased survival and reproductive parameters (Cattau et al., 2016), reflecting temporal shifts in *P. maculata* abundance following invasion (Appendix S1: Figure S1), and (2) resulted in temporal variation in source‐sink status of populations, including an increase in source populations. For long‐term snail kite population dynamics, we predicted that local dynamics (e.g., self‐recruitment, retention) played a larger role earlier in the invasion and that contributions of local populations to the other populations via emigration (i.e., the landscape contributions) increased later in the invasion.

## METHODS

### Study area and focal species

**FIGURE 2:**
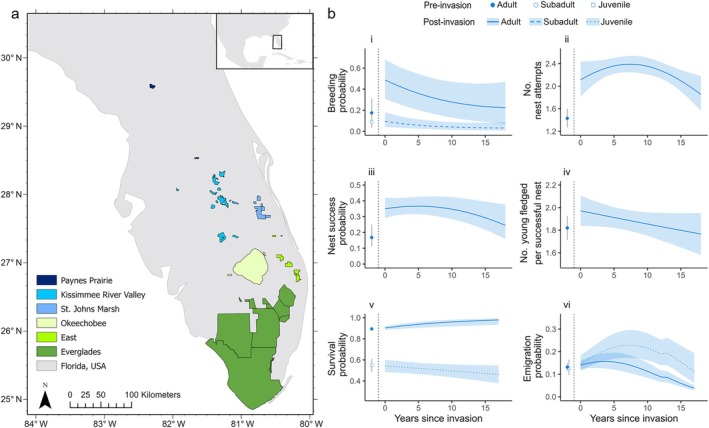
(a) Snail kite breeding habitat in Florida, USA, divided into six populations based on hydrology and geographic location. (b) Snail kite demographic rates as a function of time since invasion of *Pomacea maculata*. Shown are marginal predictions of parameters (and associated 95% uncertainty intervals) describing (i) breeding probability, (ii) number of nest attempts, (iii) nest success probability, (iv) number of young fledged per successful nest, (v) survival probability, and (vi) emigration probability, modeled without effects of year or population. Pre‐ and post‐invasion are to the left and right of the vertical dashed line, respectively. The Kissimmee River Valley is used as an example population for the emigration panel. The bump in emigration is when Paynes Prairie became available as a population to emigrate to. The relationship between nest success probability and time since invasion is not significant.

The Everglade snail kite forms a spatially structured population limited year‐round to flooded freshwater wetlands and shallow lakes in peninsular Florida, USA. Wetlands are temporally dynamic in quality due to changes in hydrology, which can alter the availability of prey and nesting substrates (Cattau et al., 2014; Darby et al., 2008, 2012; Appendix S1: Section S1). Our study included all known breeding areas for the Everglade snail kite (Figure 2a). Wetlands were grouped into six regional populations based on location and hydrology: Paynes Prairie, Kissimmee River Valley, Okeechobee, Everglades, East, and St. Johns Marsh (Appendix S1: Table S1); all were separated by upland habitat unsuitable for snail kites.

Snail kites are dietary specialists that feed almost exclusively on *Pomacea* spp. Historically, snail kites in the United States were limited to a single native apple snail species (*Pomacea paludosa*) (Sykes, 1987), but recently they have consumed the invasive non‐native *P. maculata*, established in Florida in the 1990s (Horgan et al., 2014; Rawlings et al., 2007). Locations became invaded in different years (Appendix S1: Table S1), so the length of time since invasion varied by population. See Appendix S1: Section S2 for additional information on *P. maculata* and population invasion status.

We used data on snail kite reproduction, survival, and movement from all major wetlands that snail kites used for breeding between 1996 and 2023. Snail kites were monitored each year during the peak breeding season using a systematic survey design covering the full extent of their range, including all current and historical breeding sites (Dreitz et al., 2002; Martin et al., 2007). Each wetland within a population was surveyed via airboat every 18 to 21 days between March 1 and June 30 (the peak of the breeding season; 4 to 6 surveys each year) and during monthly “preseason” surveys in January (2008, 2011–2023) and February (2003, 2008, 2011–2023). “Postseason” surveys took place in October (2009–2012, 2014–2023) in more recent years. During surveys, all observed snail kites were counted, individuals with color bands were resighted, and attempts were made to find all nests. Additional surveys were conducted throughout the year to monitor active nests and confirm breeding where snail kites were observed. Nests were visited every 2 to 3 weeks until nests failed or young successfully fledged (~24 days old). All nestlings >16 days old were banded before fledging. At each nest visit, we identified banded adults present.

### Fecundity

We assessed source‐sink dynamics using estimates of fecundity, movement, and survival (Furrer & Pasinelli, 2016; Runge et al., 2006). Fecundity, $M_{\mathrm{rta}}$, was the expected number of females fledged per adult female in population *r* in year *t* at age *a* (juvenile = 0 [<1 year old], subadult = 1 [1–2 years old], adult = 2 [>2 years old]). We included age because subadults were expected to have a lower breeding probability than adults (Reichert et al., 2012). Juveniles were assumed not to breed (i.e., $M_{\mathrm{rt}0}=0$). We estimated fecundity as

(1) $$M_{\mathrm{rta}}=\theta_{\mathrm{rta}}\times N_{\mathrm{rt}}\times P_{\mathrm{rt}}\times Y_{\mathrm{rt}},$$

where $\theta_{\mathrm{rta}}$ was the breeding probability for individuals, $N_{\mathrm{rt}}$ was the number of nesting attempts given breeding, $P_{\mathrm{rt}}$ was the probability of nest success, and $Y_{\mathrm{rt}}$ was the number of female young fledged per successful nest (assuming a 1:1 sex ratio where half of fledglings are female) (Beissinger, 1995; Martin et al., 2008).

We estimated breeding probability and the number of nesting attempts per individual using long‐term mark‐resight and nest monitoring data. These data included 2818 individually marked snail kites that were observed at least once as a subadult or adult and 2387 nests that had at least one marked adult observed at the nest. We used a hierarchical model, analyzed in a Bayesian framework, with two different components: one to estimate the breeding state and detection probability at the nests, and another to model the number of nesting attempts, conditional on breeding. See Appendix S1: Section S3 for a full model description. We analyzed our model in R (version 4.3.1) (R Core Team, 2024) and JAGS (version 4.4.1) using the “jagsUI” package (Kellner & Meredith, 2024). We ran three chains for 150,000 Markov chain Monte Carlo (MCMC) iterations and thinned chains by 50 after a burn‐in of 5000 and an adaptation phase of 1000. We assessed model convergence using the Gelman‐Rubin R‐hat statistic. All R‐hat values were <1.05, indicating successful algorithm convergence (Gelman & Hill, 2007).

We used nest monitoring data from 6990 nests to estimate the probability of nest success and the number of female young fledged per successful nest (2789 nests). We did not expect nest success or the number of young fledged to vary by age, as both strongly depend on external factors (e.g., water levels, prey density) (Cattau et al., 2014; Fletcher et al., 2021). For the probability of nest success, we used a complementary log–log regression to estimate the nest daily mortality rate, where exposure days (i.e., the time between nest checks) were considered via an offset (Fletcher et al., 2021; Heisey et al., 2007). We back‐transformed estimates to focus on nest survival. We estimated nest success by raising the daily survival rate to the power of the length of the nesting cycle (~58 days). Models for the number of young fledged were fit using a log‐link function, assuming a Poisson distribution. We fit both models using generalized linear mixed models in R with the “glmmTMB” package (version 1.1.9) (Brooks et al., 2025) and included random intercepts of population and year.

### Survival and movement

We used mark‐resight information from 5682 individually marked snail kites to estimate annual survival ($S_{\mathrm{rta}}$) and movement probabilities. We expected adults and subadults to have comparable survival and movement probabilities (i.e., $S_{\mathrm{rt}1}=S_{\mathrm{rt}2}$) but included age because juveniles have lower survival (Bennetts et al., 1999). Movement probabilities included fidelity and emigration. Emigration probability was the sum of all individual movement probabilities ($\psi_{\text{rsta}}$) from a current population *r* to a different population *s* from year *t* to *t* + 1:

(2) $$1-\psi_{\text{rrta}}=\sum_{s\neq r}\psi_{\text{rsta}},$$

where $\psi_{\text{rrta}}$ was fidelity. We used a multistate survival model (Schwarz et al., 1993) constructed using the RMark interface (version 3.0.0) (Laake, 2013) and fit by MARK (version 6.2) (White & Burnham, 1999). See Appendix S1: Section S4; Figure S2 for details on model construction.

### Time since invasion

For each vital rate, in addition to population‐level and year‐specific effects included in each model, we tested linear, quadratic, and logarithmic (covariates were log‐transformed) effects of years since *P. maculata* invasion. We tested for linear and logarithmic effects instead of exponential or logistic curves because *P. maculata* colonization and the resulting boost in food supply fostered rapid local increases in snail kite nesting, with minimal time lags (e.g., when new wetlands were created via management, high densities of nests have occurred within 6 months of wetland creation; Appendix S1: Table S2; Figure S3). We tested for interactive effects between years since invasion and age in the survival and movement models because invasion may have affected younger birds differently (Cattau et al., 2016; Poli et al., 2022). We ranked models for each vital rate based on corrected Akaike information criterion for small samples (AIC_c_; Burnham & Anderson, 2004) or Deviance information criterion for Bayesian models (DIC; Spiegelhalter et al., 2002) (Appendix S1: Tables S3–S5). Fecundity, survival, and movement estimates for the source‐sink assessment came from the top‐ranked model for each vital rate. Parameter estimates were considered significant if the 95% credible intervals or CIs did not overlap zero.

To compare pre‐ and post‐invasion scenarios, a preinvasion value was estimated for each vital rate using data prior to invasion. Preinvasion values were estimated separately to estimate the distinct relationship between vital rates and time since invasion, and because we expected patterns in vital rates before the invasion to differ from those after the invasion (Cattau et al., 2016; Poli et al., 2022; Reichert et al., 2021). Note that the Paynes Prairie population had no breeding snail kites until after the *P. maculata* invasion, and therefore this population lacked preinvasion data.

### Source‐sink dynamics

We used a source‐sink metric of the per capita contribution of a member of local population *r* to the metapopulation, $C_{r}$, proposed by Runge et al. (2006) (Figure 1a). The $C_{r}$ metric combines retention and emigration to assess both how a local population maintains itself through self‐recruitment and contributes to other populations via emigration. It provides a measure over a single time step comparable to reproductive value over long time periods (Runge et al., 2006). A population with $C_{r}>1$ is a source, $C_{r}=1$ is stable, and $C_{r}<1$ is a sink. The literature contains other definitions of sources and sinks (Loreau et al., 2013; Runge et al., 2006), but the $C_{r}$ metric helps assess the role of populations in metapopulations of highly mobile species, such as the snail kite.

We expanded $C_{r}$ to include variation in population contributions by year *t*. $C_{\mathrm{rt}}$ was estimated as:

(3) $$C_{\mathrm{rt}}=\frac{N_{t2}}{N_{t}}\left(C_{\mathrm{rt}2}\right)+\frac{N_{t1}}{N_{t}}\left(C_{\mathrm{rt}1}\right)$$

where $\frac{N_{\mathrm{ta}}}{N_{t}}$ was the proportion of the population in year *t* in age class *a*, and $C_{\mathrm{rta}}$ was the per capita contribution for each age class (juvenile = 0 [<1 year old], subadult = 1 [1–2 years old], adult = 2 [>2 years old]). We determined adult and subadult proportions using the raw proportion of banded snail kites observed within a year in each age class. The per capita contribution for each age group was

(4) $$\begin{array}{l} C_{\mathrm{rta}}=\overset{\text{Adult}\;\text{retention}}{\overbrace{\phi_{\text{rrta}}}}+\overset{\text{Adult}\;\text{emigration}}{\overbrace{\sum_{S\neq r}\phi_{\text{rsta}}}}\\ \;+\underset{\text{Fecundity}}{\underbrace{M_{\mathrm{rta}}}}\left(\overset{\text{Juvenile}\;\text{retention}}{\overbrace{\phi_{\mathrm{rrt}0}}}+\underset{\text{Juvenile}\;\text{emigration}}{\underbrace{\sum_{S\neq r}\phi_{\mathrm{rst}0}}}\right). \end{array}$$

Here, $\phi_{\text{rrta}}$ (retention) was the joint probability of both surviving ($S_{\mathrm{rta}}$) and remaining in population *r* from year *t* to *t + 1* ($\psi_{\text{rrta}}$) and $\phi_{\text{rsta}}$ (emigration) was the joint probability of both surviving ($S_{\mathrm{rta}}$) and moving from population *r* to a different population *s* ($\psi_{\text{rsta}}$). Estimates of joint movement and survival probabilities were equal for adults and subadults whereas estimates of fecundity were expected to differ ($M_{\mathrm{rt}2}\neq M_{\mathrm{rt}1}$). Note that survival was the sum of retention and emigration ($S_{\mathrm{rta}}=\phi_{\text{rrta}}+\sum_{s\neq r}\phi_{\text{rsta}}$) and Equation (4) reduced to $C_{\mathrm{rta}}=S_{\mathrm{rta}}+M_{\mathrm{rta}}S_{\mathrm{rt}0}$. In a spatially closed population this is the finite rate of increase.

The separation of the joint probabilities of survival and fidelity ($\phi_{\text{rrta}}$) and survival and emigration ($\phi_{\text{rsta}}$) in Equation (4) was necessary to calculate a closely related metric: $R_{\mathrm{rt}}$. $R_{\mathrm{rt}}$ is the ability of a local population to maintain itself through self‐recruitment and retention (Runge et al., 2006). The equation for $R_{\mathrm{rt}}$ for each age group is similar to $C_{\mathrm{rt}}$ but only includes terms for retention and self‐recruitment:

(5) $$R_{\mathrm{rta}}=\phi_{\text{rrta}}+M_{\mathrm{rta}}\phi_{\mathrm{rrt}0}.$$

$C_{\mathrm{rt}}$ and $R_{\mathrm{rt}}$ can help identify how populations differ in their contribution to local versus metapopulation growth. $R_{\mathrm{rt}}$ is related to $C_{\mathrm{rt}}$ by

(6) $$R_{\mathrm{rt}}=C_{\mathrm{rt}}-E_{\mathrm{rt}},$$

where $E_{\mathrm{rt}}$ is the emigration rate from population *r* (i.e., the landscape contribution of population *r* to other populations in the metapopulation; $E_{\mathrm{rta}}=\sum_{s\neq r}\left(\phi_{\text{rsta}}+M_{\mathrm{rta}}\phi_{\mathrm{rst}0}\right)$). If $R_{\mathrm{rt}}=C_{\mathrm{rt}}$, population *r* produced no emigrants in year *t* and all surviving young were recruited into the local population. If $C_{\mathrm{rt}}>1$, but $R_{\mathrm{rt}}<1$, the population was a net exporter but would not be self‐sustaining if such patterns continued due to high emigration (Runge et al., 2006). This type of population is sometimes named a “dependent source” (Hixon et al., 2002; see also “conditional source” Loreau et al., 2013).

We used the mean and SE of each fecundity, movement, and survival parameter in a stochastic simulation (Morris & Doak, 2002) with 10,000 replicates to estimate uncertainty in our $C_{\mathrm{rt}}$, $R_{\mathrm{rt}}$, and $E_{\mathrm{rt}}$ estimates. Replicates for mean survival, movement, nest success, and breeding probability were all sampled assuming beta distributions because probabilities are real numbers between zero and one. Replicates for mean number of nesting attempts and mean number of young produced were sampled assuming a log‐normal distribution. The 0.025 and 0.975 quantiles were calculated from the replicates to produce 95% CIs on $C_{\mathrm{rt}}$. Populations were considered a source in year *t* when these quantiles were >1, a sink when quantiles were <1, and stable when quantiles overlapped one (Runge et al., 2006).

## RESULTS

### Vital rates and time since invasion

Time since *P. maculata* invasion had significant relationships with breeding probability, the number of nest attempts, the number of young fledged per successful nest, survival, and movement, based on the most‐supported models (Figure 2b, Appendix S1: Tables S3–S5). This indicates that the *P. maculata* invasion influenced vital rates in addition to population‐level or year‐specific effects (e.g., annual variation in drought conditions) also included in all models. Survival probability and the number of young fledged were linearly related to time since invasion (Figure 2b‐iv,v), whereas breeding probability, the number of nest attempts, and emigration probability had quadratic relationships (Figure 2b‐i,ii,vi). We found no evidence for logarithmic relationships.

Adult breeding probability increased post‐invasion (${\bar{\theta }}_{t=0,a=2}=0.51\pm 0.15\;\mathrm{SD}$) and then declined toward preinvasion estimates in later years (${\bar{\theta }}_{t=8,a=2}=0.34\pm 0.18\;\mathrm{SD}$) ($\beta_{\text{time}}=-1.24$, 95% CI [−2.10, −0.40]; $\beta_{\mathrm{tim}e^{2}}=0.29$, 95% CI [−0.05, 0.64]) (Figure 2b‐i). Subadult breeding probability tended to be lower ($\theta_{a=1}<0.24$) than adult breeding probability. The number of nest attempts were higher post‐ than preinvasion and increased and subsequently decreased slightly with increasing time since invasion ($\beta_{\text{time}}=0.34$, 95% CI [−0.01, 0.70]; $\beta_{\mathrm{tim}e^{2}}=-0.23$, 95% CI [−0.42, −0.03]) (Figure 2b‐ii). Similarly, nest survival was higher post‐ than preinvasion, but CIs for the parameters representing the time since invasion effect overlapped zero ($\beta_{\text{time}}=6.71$, 95% CI [−1.94, 15.36]; $\beta_{\mathrm{tim}e^{2}}=4.69$, 95% CI [−0.06, 9.45]) (Figure 2b‐iii). The negative relationship between the number of young fledged and time since invasion was weak ($\beta_{\text{time}}=-0.01$, 95% CI [−0.01, 0.00]) (Figure 2b‐iv).

Survival had a positive relationship with time since *P. maculata* invasion ($\beta_{\text{time}}=0.10$, 95% CI [0.03, 0.16]), but the interactive effect between time since invasion and age was weakly negative for juveniles ($\beta_{\text{time},a=0}=-0.02$, 95% CI [−0.04, 0.00]) (Figure 2b‐v) (see Appendix S1: Figure S4 for population‐ and year‐specific estimates). Emigration probability had a quadratic relationship with time since invasion, increasing and then decreasing ($\beta_{\text{time}}=0.09$, 95% CI [0.03, 0.15]; $\beta_{\mathrm{tim}e^{2}}=-0.01$, 95% CI [−0.01, −0.01]) (Figure 2b‐vi). The Kissimmee River Valley lacked sufficient data to estimate movement in 2006 and 2008 and was omitted from the source‐sink analysis in those years.

### Sources, sinks, and invasion

**FIGURE 3:**
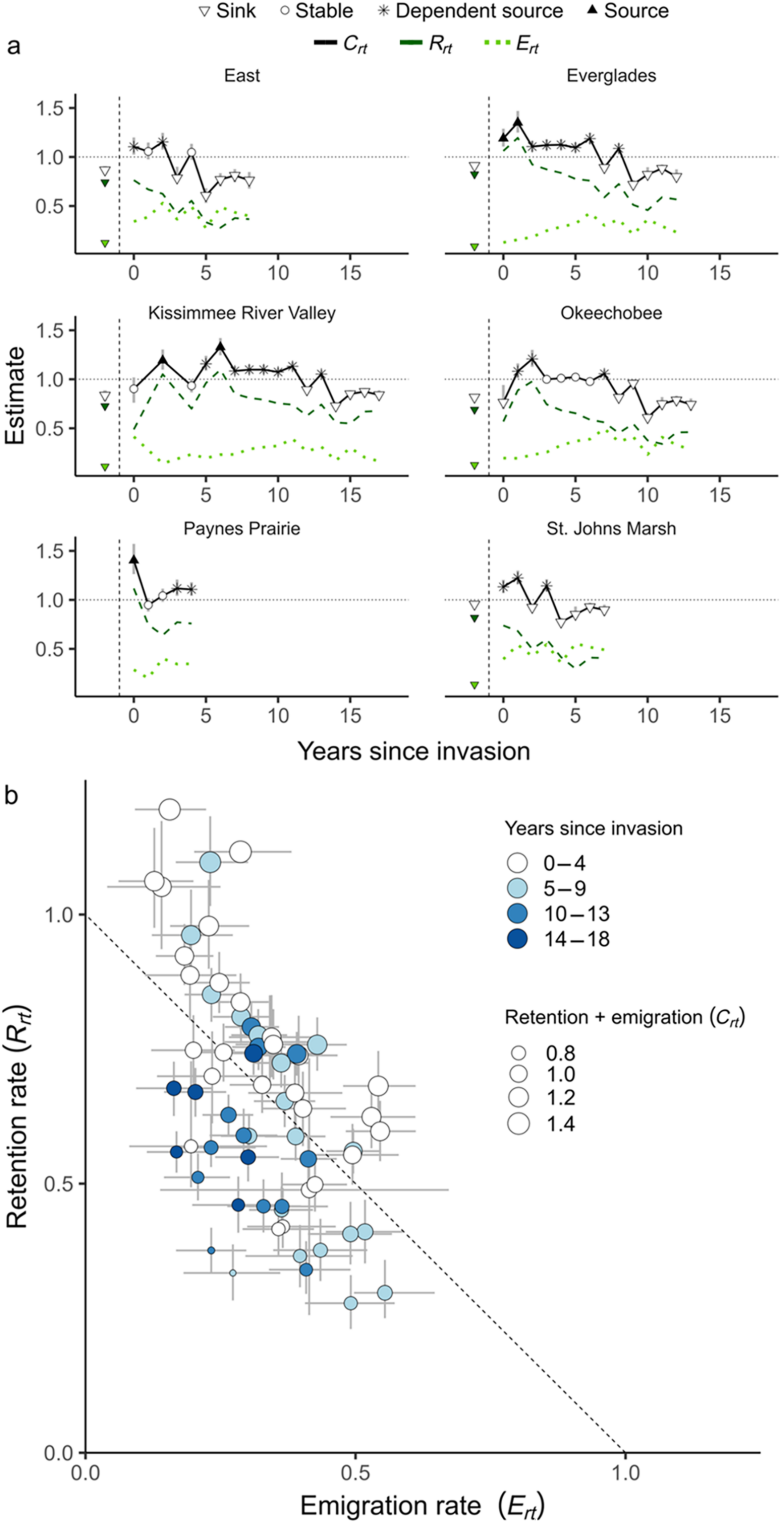
(a) Source‐sink estimates ($C_{\mathrm{rt}}$), local recruitment and retention ($R_{\mathrm{rt}}$), and emigration rate ($E_{\mathrm{rt}}$) for six snail kite populations as a function of years since *Pomacea maculata* invasion in each population. $E_{\mathrm{rt}}$ incorporates the survival and nonlocal recruitment of individuals that emigrate from population *r*. A larger $R_{\mathrm{rt}}$ indicates greater retention of individuals into the local population. A larger $E_{\mathrm{rt}}$ indicates a greater contribution of individuals from a local population to the landscape through emigrants. Pre‐ and post‐invasion are to the left and right of the vertical dashed line, respectively. Gray bars represent 95% CIs on $C_{\mathrm{rt}}$. We define sources, sinks, and stable populations based on the 95% CIs of $C_{\mathrm{rt}}$ (sources: 95% $C_{\mathrm{rt}}>1$, sinks: 95% $C_{\mathrm{rt}}<1$, stable: 95% $C_{\mathrm{rt}}$ overlaps 1). Dependent sources are net exporters of individuals but would not be self‐sustaining due to high rates of emigration if such patterns continued over time. Preinvasion estimates include all the years before a population was invaded, beginning in 1996. Populations became invaded in different years and therefore the length of the time series of years since invasion varies by population. (b) Population‐specific estimates of $R_{\mathrm{rt}}$ as a function of $E_{\mathrm{rt}}$ colored by years since *P. maculata* invasion and sized by the per capita contribution of a member of local population *r* to the metapopulation $\left(C_{\mathrm{rt}}\right)$. Gray bars represent 95% CIs on $E_{\mathrm{rt}}$ and $R_{\mathrm{rt}}$. Below the dashed line are sinks or stable populations with $C_{\mathrm{rt}}<1$ and above are sources or stable population with $C_{\mathrm{rt}}>1.$

Source‐sink status fluctuated widely with time since invasion (Figure 3a). Before the *P. maculata* invasion, all populations were sinks with $C_{\mathrm{rt}}<1$. However, no population was consistently a source or sink post‐invasion; populations were a source or stable with $C_{\mathrm{rt}}\geq 1$ approximately half (51%) of the time, on average, across all populations (Appendix S1: Figure S5). Source or stable populations with $C_{\mathrm{rt}}\geq 1$ were more common early in the invasion ($\bar{x}=4.2\;\text{years}\;\text{since invasion}\;\pm 3.4\;\text{SD}\;$), as compared to sinks ($\bar{x}=7.9\;\text{years}\;\text{since invasion}\;\pm 4.6\;\text{SD}$).

We predicted $R_{\mathrm{rt}}$ would be highest early in the invasion and $E_{\mathrm{rt}}$ would increase later. Our results supported our predictions: we found higher $R_{\mathrm{rt}}$ occurred early in the invasion, whereas $E_{\mathrm{rt}}$ tended to peak later in the invasion (Figure 3b). The average years since invasion when $R_{\mathrm{rt}}>1$ was 1.8 years ±2.5 SD. Patterns varied by population, but $R_{\mathrm{rt}}$ often declined over time and dipped below $E_{\mathrm{rt}}$ in a few cases (e.g., East, Okeechobee, St. Johns Marsh; Figure 3a). Source and stable populations with $C_{\mathrm{rt}}\geq 1$ were associated with greater local recruitment and retention $\left(R_{\mathrm{rt}}\right)$, on average, than sinks and stable populations with $C_{\mathrm{rt}}<1$ ($\bar{R_{\mathrm{rt}}}=0.50\pm 0.13\;\mathrm{SD}$ when $C_{\mathrm{rt}}<1$; $\bar{R_{\mathrm{rt}}}=0.81\pm 0.17\;\mathrm{SD}$ when $C_{\mathrm{rt}}\geq 1$), whereas emigration ($E_{\mathrm{rt}}$) was generally the same on average ($\bar{E_{\mathrm{rt}}}=0.33\pm 0.11\;\mathrm{SD}$ when $C_{\mathrm{rt}}<1$; $\bar{E_{\mathrm{rt}}}=0.33\pm 0.11\;\mathrm{SD}$ when $C_{\mathrm{rt}}\geq 1$).

Of the 33 occasions when populations were sources or stable with $C_{\mathrm{rt}}>1$, 70% were estimated with $C_{\mathrm{rt}}>1$ and $R_{\mathrm{rt}}<1$ (i.e., a dependent source). This result has two important implications. First, it suggests that if such patterns continued over time populations would not be self‐sustaining due to high emigration. Second, it suggests that sources largely arise from high landscape contributions rather than local contributions.

## DISCUSSION

Empirical investigations of temporal variation in source‐sink dynamics are important for relating metapopulation theory to the dynamics of wild populations and for guiding appropriate conservation and management strategies aimed at increasing (meta)population growth. We found that snail kite populations underwent striking changes in source‐sink dynamics over time, concordant with time since invasion of the non‐native *P. maculata* apple snail. Over time, no population was consistently a sink or a source. Our results also reveal how an invasive species can cause effects outside of invaded areas due to changing emigration rates of native species. Fluctuating local retention and emigration rates fundamentally changed the interpretation of source‐sink dynamics through variation in the local‐ versus landscape‐scale contribution of populations to the metapopulation over time. We discuss these patterns and their implications for understanding long‐term source‐sink dynamics and for conservation.

Invasive species that provide resources can benefit native consumers (Pintor & Byers, 2015). Benefits of the *P. maculata* invasion for snail kites are well documented, including rapid increases in juvenile survival and the number of young fledged, longer breeding seasons, and increased body mass (Cattau et al., 2016, 2018; Fletcher et al., 2024; Poli et al., 2022). However, by tracking long‐term changes in vital rates we found that some positive effects of the *P. maculata* invasion diminished over time (Figure 2b). For example, early increases in breeding probability returned to near preinvasion estimates after 15 years. This decline could either result from nonlinear changes in *P. maculata* abundance (e.g., boom‐bust dynamics, Appendix S1: Figure S1; Strayer et al., 2017) with increasing time since invasion, or from density dependence (Amarasekare, 2004) in snail kite vital rates. However, local snail kite population counts in the previous year showed no strong relationship with $C_{\mathrm{rt}}$ (Appendix S1: Figure S6), suggesting density dependence overall was weak when combined across vital rates. Our results highlight how long‐term effects of invasions may take decades to fully realize.

Invasive species can both directly and indirectly impact native species demography (Gallardo et al., 2016; Rodriguez, 2006; Stodola et al., 2013) and thus alter the source‐sink status of native populations (Lejeune et al., 2023; Woodford & McIntosh, 2010). As predicted, local recruitment and retention ($R_{\mathrm{rt}}$) was highest early in the invasion, during a time of rapid increase in reproductive potential (Figure 3b; Cattau et al., 2016). The contribution of a local population to other populations (via emigration, $E_{\mathrm{rt}}$) peaked slightly later in the invasion. Over the long‐term, populations were sources shortly after they were invaded by *P. maculata* and became sinks in later years. Variation in the year a population became invaded (Appendix S1: Table S1) meant increased emigration from the first invaded populations boosted uninvaded populations. These results illustrate an important mechanism through which invasive species can cause impacts in uninvaded populations.

Fluctuating retention and emigration rates alter source‐sink dynamics by tipping the scale of local‐ and landscape‐scale contributions by each population to the metapopulation. Consider two populations with a per capita metapopulation contribution ($C_{r}$) of 1.1. These populations would typically be considered sources. Yet, if one population has an emigration rate $\left(E_{r}\right)$ of 0.4 and the other $E_{r}=0.1$, these populations would have different implications for source‐sink dynamics. The first would provide greater landscape‐scale contributions, but risks local population decline, whereas the second would be locally stable but contributes less at the landscape scale (Figure 1b). When comparing $C_{\mathrm{rt}}$ and $R_{\mathrm{rt}}$ in snail kites long‐term, we found 70% of source or stable populations with $C_{\mathrm{rt}}\geq 1$ would not be self‐sustaining through local recruitment and retention ($R_{\mathrm{rt}}<1$) if such patterns continued. The frequency of these “dependent” (Hixon et al., 2002; Runge et al., 2006) source populations demonstrates the importance of landscape‐scale contributions to snail kite metapopulation persistence. Our results highlight how contrasting retention and emigration over time can increase understanding of local‐ and landscape‐scale contributions to long‐term metapopulation dynamics.

Information about individual movements is necessary to accurately characterize source‐sink dynamics (Furrer & Pasinelli, 2016; Paquet et al., 2020). Without assessing emigration, most snail kite populations would have appeared to be sinks due to high loss rates (the sum of emigration and mortality) (Figure 3a). High rates of emigration from sources can occur for several reasons. For instance, density dependence can force emigration of individuals into lower quality habitat (Pulliam, 1988), avoidance of inbreeding or kin competition can lead to high emigration (Hamilton & May, 1977), or a metapopulation with ephemeral habitats can have high emigration rates (Diffendorfer, 1998). Yet, emigration is challenging to estimate in many systems, given it requires reliably tracking individuals and distinguishing permanent emigration from mortality. This is likely why emigration is rarely included in source‐sink studies (Furrer & Pasinelli, 2016; Heinrichs, Walker, et al., 2019). Emigration may be less important for assessing source‐sink status in the short term for metapopulations where extinctions are driven by local processes. However, information about emigration is required when local patch extinction is determined more by movement than local mortality (Harrison, 1991; Reigada et al., 2015). In transient or disturbance‐prone populations with high emigration rates, such as pond‐breeding amphibians (Peterman et al., 2013), or symbionts that abandon hosts when death is threatened (Mestre et al., 2021), source‐sink designations without detailed information on individual movement may be misleading.

Populations can fluctuate in source‐sink status over time with changes in habitat quality (Heinrichs, Lawler, et al., 2019). We identified temporally varying source‐sink dynamics in snail kites, reflecting changes in vital rates during the *P. maculata* invasion. No population was consistently a sink or source post‐invasion. Consequently, short‐term snapshots of source‐sink status may not reflect long‐term variation in dynamics in some systems. Yet, empirical examples of fluctuating source‐sink status in wild populations are rare (e.g., Badia‐Boher et al., 2025; Johnson, 2004; Walker et al., 2016), and conservation has historically prioritized identifying, presumably constant, source populations (e.g., Kirol et al., 2015; Margules & Pressey, 2000). Future work that examines source‐sink dynamics at multiple spatial scales in environments undergoing shifts in habitat quality (e.g., due to habitat degradation, biological invasions) could be used to evaluate source‐sink constancy.

Our work emphasizes the importance of long‐term studies rather than snapshots for identifying which spatial components are of greatest potential conservation value for threatened species. The insight that the Everglade snail kite lacks a constant source population would have been difficult or impossible to confirm in a short‐term study. Maintaining a network of local populations, including sink populations, can increase metapopulation persistence by providing additional breeding habitat (Pulliam, 1988) and by providing opportunities for individuals to exploit spatiotemporal variability in fitness across landscapes (Kortessis et al., 2025). We suggest that, where possible, future research should employ a landscape perspective on the pattern of source‐sink status over time to evaluate populations of threatened species (Loreau et al., 2013). Expanding research into two or more populations can provide important inference not discernible from a single population (e.g., Paquet et al., 2020; Weegman et al., 2016). Source constancy can be used to prioritize areas for protection or restoration and, critically, evaluate trade‐offs in spreading resources across multiple populations versus implementing protective measures in a single or few populations. In cases where expanding the spatial or temporal scope of research is not possible, collecting ancillary information (e.g., tracking movements of individuals, using genetic data) could assist in identifying the importance of local versus landscape contributions in a metapopulation.

## AUTHOR CONTRIBUTIONS

Meghan A. Beatty and Robert. J. Fletcher Jr conceived the study. Meghan A. Beatty and Robert. J. Fletcher Jr collected data. Meghan A. Beatty, Robert. J. Fletcher Jr, Ismael V. Brack, and Denis Valle contributed to model design. Meghan A. Beatty and Robert. J. Fletcher Jr analyzed data. Meghan A. Beatty wrote the first draft of the manuscript. All authors contributed substantial effort in ideas and revisions of the manuscript.

## CONFLICT OF INTEREST STATEMENT

The authors declare no conflicts of interest.

## Supporting information


Appendix S1.

## Data Availability

Data and code (Beatty, 2024) are available in Figshare at https://doi.org/10.6084/m9.figshare.c.7067789.v5.
