# Supplementary material for: An invasive prey alters local and landscape contributions of sources and sinks for an endangered predator
Source: Ecology. 2025 Nov 16;106(11):e70242. doi: 10.1002/ecy.70242 (PMC12620111; doi:10.1002/ecy.70242)
Supplement: Supplementary file 1 — Appendix S1. [file ECY-106-e70242-s001.pdf]

APPENDIX S1

An invasive prey alters local and landscape contributions of sources and sinks for an endangered predator

Meghan A. Beatty, Ismael V. Brack, Robert D. Holt, Denis Valle, Robert. J. Fletcher, Jr

Ecology

Section S1 - Wetland hydrology and habitat quality Ecology

We used 28 years of demographic data to understand how a novel invasive prey species altered snail kite vital rates and source-dynamics in the long-term. However, there are additional environmental factors that could affect short-term (e.g., annual, seasonal) dynamics. Wetlands used by snail kites can change over time due to short-term fluctuations in water levels (Fletcher et al. 2021). Habitat management can alter the availability of vegetation substrates for nesting (Benscoter et al. 2023, Gonzalez and Fletcher 2025) and foraging (Bennetts et al. 2006). Water levels are strictly controlled in most wetlands by different management agencies (South Florida Water Management District [SFWMD] 2023). Vegetation that provides nesting substrate (e.g., willows, cattail) (Snyder et al. 1989) or floating vegetation that can obscure snails (e.g., water hyacinth) can be removed or modified through prescribed burns (Benscoter et al. 2023) or herbicide treatments (Florida Fish and Wildlife Conservation Commission [FWC] 2024). Thus, habitat change can affect snail kites directly by changing available nesting substrates or indirectly by altering apple snail availability. Annual fluctuations in source-sink status could also reflect short-term changes in habitat quality driven by climate and wetland hydrology which can rapidly impact snail kite vital rates (Beissinger 1995, Mooij et al. 2002, Fletcher et al. 2021).

Section S2 - Pomacea maculata and time since invasion

Pomacea maculata is larger (Darby et al. 2007), more fecund (Kyle et al. 2013), and has a greater drought tolerance (Glasheen et al. 2017) than the native apple snail (*Pomacea paludosa*). *P. maculata* first invaded a wetland used by snail kites in 2005 (Cattau et al. 2010) and spread across the vast majority of the snail kite's range by 2012 (Poli et al. 2022). Prior to 2010 invasion status for each population and year was determined using snail shells collected from snail kite feeding perches and nests in addition to snail surveys conducted by the Florida Fish and Wildlife Conservation Commission (FWC unpublished data; Cattau et al. 2016). After 2010 invasion status for each population and year was determined using *P. maculata* occurrence data collected during systematic snail kite surveys (see *Data Collection*). A population was considered invaded when *P. maculata* was detected during at least 50% of systematic surveys and snail kite nesting occurred.

Section S3 – Breeding probability and the number of nest attempts

We estimated breeding probability and the number of nesting attempts per individual using long-term mark-resight and nest monitoring data. We used a hierarchical model, analyzed in a Bayesian framework, with two different components, one to estimate the breeding state and detection probability at the nests, and another to model the number of nesting attempts, conditional on breeding.

For the first part of the model, we assumed that each individual *i,* in population *r*, in year *t*, at age *a,* is breeding or not with a probability θ_{rta} so that:

$$z_{irta} \sim Bernoulli(\theta_{rta}),$$

in which z_{irta} is the latent binary state indicating if the individual is breeding or not breeding. We modeled θ_{rta} as a function of age (A_{irt}) and random intercepts for year and population:

$$logit(\theta_{rta}) = \gamma_{0t} + \gamma_{1r} + \gamma_2 I(A_{irt} = 2).$$

We assumed that the number of times where an individual was detected breeding (i.e., re-sighted at the nest), b_{irta}, out of the total number of detections, d_{irta}, came from a binomial distribution:

$$b_{irta} \sim Binomial(p_r z_{irta},\ d_{irta}),$$

where p_r is the detection probability of an adult at a nest, given the nest was detected. We modeled detection probability as constant.

The second part of the model is focused on number of nesting attempts, conditional on breeding. We assume that each breeding individual ($z_{irta} = 1$) attempted to breed N_{rt} times with an expected mean λ, so that:

$$N_{rt} \sim Poisson(z_{irta}\lambda).$$

λ was considered as constant because parameters were poorly estimated when random effects of year and population were included. Furthermore, when we ran the model with random effects we obtained similar results. For the observation level, we assumed that the observed number of nesting attempts n_{irt} came from a binomial distribution:

$$n_{irt} \sim Binomial(p_r\ , N_{irt}),$$

where p_r is taken from the first part of the model.

We chose uninformative priors for all fixed effect coefficients (i.e., N(0, 100)). We chose a Uniform prior distribution for λ where:

$$\lambda \sim \mathrm{U}(0, 6).$$

We modeled random effects by specifying hyperpriors drawn from a normal distribution, N (μ, σ^2) where:

$$\mu \sim N(0, 100)$$

$$\sigma \sim U(0,10)$$

Section S4 - Survival and movement

We used a multi-state survival model (Schwarz et al. 1993) constructed using the RMark interface (Laake 2013) and fit by MARK (White and Burnham 1999). Multi-state models require making assumptions on whether individuals survive and then move or move and then survive. We assumed survival occurred before movement and therefore survival probabilities were the same regardless of whether an individual emigrated or not. This is a reasonable assumption given that snail kites have a strong dispersal ability (Reichert et al. 2021) and juvenile survival depends on natal location (Poli et al. 2024).

We constructed encounter histories with one occasion per year using resights from all surveys (1996-2023). We included resights between surveys collected during nest monitoring activities. We modeled detection probability by wetland type (lacustrine or palustrine; Fletcher Jr. et al. 2015). All survival and movement components of the multi-state models included fixed effects of year to account for unexplained annual variation. Including time since invasion and year in the same model could result in partial confounding but we did not expect this to be the case as populations have different time since invasion values in a given year. Further, time since invasion was modeled as a continuous covariate, in contrast to year, which was modeled as a categorical covariate. The survival component of the model included a fixed effect of population.

Movement was estimated as a function of distance between centroids of populations to account for decreased movement probability with increasing distance (Fletcher Jr. et al. 2011, 2013) (Fig. S2) and to improve estimability of parameters.

Table S1**.** Wetlands and impoundments with snail kite nesting were divided into six populations based on geographic location (Fig. S3) and hydrology. Each population encompasses one or two hydrologic units (i.e., watersheds or pieces of watersheds). The wetlands or impoundments within each are similar in wetland type (i.e., lacustrine or palustrine). 'Year invaded' is the first year at least one wetland within the population was invaded by the non-native *Pomacea maculata* apple snail and snail kites began nesting in the invaded wetland.

Population	Wetlands/Impoundments	Year invaded
Kissimmee River Valley	East Lake Tohopekaliga; Lake Runnymede; Lake Tohopekaliga; Lake Hatchineha; Lake Jackson; Lake Marian; Tiger Lake; Lake Kissimmee; Kissimmee River Restoration Area; Lake Istokpoga; Lake Paker; Lake Smart; Rolling Meadows	2005
Okeechobee	Lake Okeechobee; Lake Hicpochee Flow Equalization Basin (FEB); Lakeside Ranch Stormwater Treatment Area (STA)	2009
St. Johns Marsh	Three Forks Conservation Area; Fellsmere Water Management Area; Mary A. Mitigation Bank; Blue Cypress/St Johns Marsh; Six Mile Restoration Area	2015
Everglades	A-1 FEB; Rotenberger Wildlife Management Area (WMA); Loxahatchee NWR (Water Conservation (WCA) 1); WCAs 2A, 2B, 3A and 3B; Everglades National Park, Big Cypress National Preserve; STAs 1E/W, 2, 3/4, 5/6	2010
East	Allapattah Flats WMA; Grassy Waters Preserve; Hobe Sound NWR; Hungryland Wildlife and Environmental Area; Loxahatchee Slough Natural Area; Ten Mile Creek; C-23 Reservoir; C-44 Impoundment	2014
Paynes Prairie	Paynes Prairie	2018

Table S2**.** Novel breeding sites used briefly by snail kites, likely due to 'booms' of non-native *Pomacea maculata* apple snail abundance and/or availability (Fig. S2; Pias et al. 2016). Snail kites readily move between wetlands to follow changes in prey availability. A reduction in water levels or increased aquatic vegetation may lead to a 'bust' in prey abundance or availability. Nests are the total number of nests initiated in each site during the years where breeding occurred. Environmental change is an event that occurred prior to snail kites breeding at the site and is assumed to be the cause of the increase in apple snail availability or abundance.

Site	Years with breeding	Nests	Environmental change	Source
Shingle Marsh	2010-2011	19	Flooded pasture	Pias et al. 2016
Mary A. Mitigation Bank	2015-2018	260	Increased water level	Fletcher et al. 2019
Ten Mile Creek	2016	37	Increased water level	Fletcher et al. 2017
Rotenberger Wildlife Management Area	2017-2019	183	Wildfire	Fletcher et al. 2019
C-23 Reservoir	2018	28	Newly inundated	Fletcher et al. 2019
A-1 FEB	2021	44	Newly inundated	Fletcher et al. 2022
C-44 Impoundment	2021-2022	93	Newly inundated	Fletcher et al. 2022
Lake Hicpochee Impoundment	2021-2022	185	Increased water level	Fletcher et al. 2022
Pierce Cattle Co.	2023	60	Flooded pasture	Fletcher et al. 2024

Table S3. Model selection for breeding probability and number of nesting attempts per individual. Time is the number of years since a wetland was invaded by *P. maculata*. All breeding probability models also contain random intercepts of population and year. Age is adult (>2 years) or subadult (1-2 years). pD is the effective number of parameters, D is deviance, DIC is the deviance information criterion, and ΔDIC is the difference in DIC between a model and the model with the lowest DIC.

	Breeding probability model	Nest attempts model	pD	D	DIC	ΔDIC
1	$time^2$ + age	$time^2$	3577.8	9977.5	13555.4	0.0
2	time + age	log(time)	3618.2	9970.9	13589.0	33.7
3	$time^2$ + age	1	3616.7	9972.3	13589.1	33.7
4	age	1	3620.3	9970.0	13590.3	35.0
5	log(time) + age	log(time)	3626.7	9976.5	13603.2	47.9
6	age	time	3647.6	9969.2	13616.9	61.5
7	time + age	1	3647.0	9970.0	13617.1	61.7
8	age	log(time)	3646.3	9971.1	13617.4	62.0
9	$time^2$ + age	time	3686.8	9971.9	13658.7	103.3
10	log(time) + age	time	3684.2	9976.5	13660.7	105.3
11	log(time) + age	1	3691.7	9975.1	13666.8	111.4
12	log(time) + age	$time^2$	3698.1	9978.8	13676.9	121.6
13	age	$time^2$	3708.6	9970.5	13679.0	123.7
14	time + age	time	3707.8	9971.8	13679.6	124.2
15	$time^2$ + age	log(time)	3716.3	9973.8	13690.1	134.7
16	time + age	$time^2$	3766.0	9972.0	13738.0	182.6

Table S4. Model selection for nest success probability and number of young fledged per successful nest. Time is the number of years since a wetland with breeding snail kites was invaded by *P. maculata*. All models also contain random intercepts of population and year. K is the number of parameters, LL is the log-likelihood, AICc is Akaike's information criteria corrected for small sample sizes, and ΔAICc is the difference in AICc between a model and the model with the lowest AICc.

	Model	K	LL	AICc	ΔAICc	AICc Weight
**Nest success**						
1	$time^2$	5	-6582.2	13174.3	0	0.53
2	time	4	-6584.0	13176.0	1.71	0.23
3	1	3	-6585.3	13176.5	2.23	0.17
4	log(time)	4	-6585.2	13178.4	4.02	0.07
**Young fledged**						
1	time	4	-3027.2	6062.4	0	0.33
2	1	3	-3028.4	6062.7	0.32	0.28
3	$time^2$	5	-3026.6	6063.3	0.85	0.22
4	log(time)	4	-3027.9	6063.8	1.35	0.17

Table S5. Model selection for survival and movement probabilities. Time is the number of years since a wetland with breeding snail kites was invaded by *P. maculata*. Age is adult (>1 year) or juvenile (<1 year). All survival models include a fixed effect of population. All movement models include the distance between centroids of populations. All models also include a fixed effect of year. Detection probability is modeled as a function of wetland type (i.e., lacustrine or palustrine). K is the number of parameters, AICc is Akaike's information criteria corrected for small sample sizes, and ΔAICc is the difference in AICc between a model and the model with the lowest AICc.

	Survival	Movement	K	Deviance	AICc	ΔAICc	AICc Weight
1	age x time	age x time^2	52	19546.6	40852.5	0.00	0.48
2	age + time	age x time^2	51	19542.4	40853.4	0.98	0.29
3	age + time^2	age x time^2	52	19551.7	40854.9	2.44	0.14
4	1	age x time^2	50	19556.0	40857.6	5.12	0.04
5	age + log(time)	age x time^2	51	19556.2	40858.7	6.19	0.02
6	age x time^2	age x time^2	54	19554.4	40858.8	6.32	0.02
7	age x log(time)	age x time^2	52	19565.6	40860.1	7.63	0.01
8	age x time^2	age + time^2	53	19566.8	40869.6	17.15	0.00
9	age + log(time)	age + time^2	50	19568.5	40871.6	19.10	0.00
10	age x log(time)	age + time^2	51	19578.1	40872.7	20.27	0.00
11	age x time	age + time^2	50	19578.0	40872.8	20.31	0.00
12	age + time	age + time^2	49	19576.0	40873.2	20.75	0.00
13	age + time^2	age + time^2	50	19588.7	40874.8	22.33	0.00
14	1	age + time^2	48	19587.7	40877.2	24.70	0.00
15	age x time^2	age x time	53	19609.4	40881.6	29.10	0.00
16	age + time	age x time	49	19607.5	40884.6	32.09	0.00
17	age x time	age x time	50	19606.3	40886.0	33.54	0.00
18	age + time^2	age x time	50	19618.0	40887.3	34.79	0.00
19	age x log(time)	age x time	51	19619.8	40887.8	35.30	0.00
20	1	age x time	48	19630.8	40888.5	36.00	0.00
21	age + log(time)	age x time	49	19629.8	40890.0	37.57	0.00
22	age x time	age + time	49	19647.1	40915.2	62.70	0.00
23	age x time^2	age + time	51	19647.8	40917.1	64.68	0.00
24	age + time	age + time	48	19653.1	40918.5	66.05	0.00
25	age + log(time)	age + time	49	19653.7	40918.7	66.23	0.00
26	age + time^2	age + time	49	19661.3	40920.7	68.21	0.00
27	1	age + time	47	19660.5	40922.1	69.63	0.00
28	age x time	age x log(time)	51	19669.7	40924.9	72.41	0.00

29	age x time^2	age x log(time)	53	19671.6	40925.7	73.24	0.00
30	age x log(time)	age + time	49	19676.6	40927.2	74.73	0.00
31	age + time	age x log(time)	50	19677.0	40928.3	75.86	0.00
32	1	age x log(time)	49	19681.5	40932.8	80.33	0.00
33	age + time^2	age x log(time)	50	19687.9	40934.9	82.46	0.00
34	age x log(time)	age x log(time)	51	19698.1	40937.5	85.01	0.00
35	age + log(time)	age x log(time)	49	19697.2	40942.6	90.11	0.00
36	age x time	age + log(time)	49	19703.9	40955.5	103.02	0.00
37	age x time^2	age + log(time)	51	19701.1	40955.6	103.18	0.00
38	1	age + log(time)	48	19708.1	40956.9	104.46	0.00
39	age + time^2	age + log(time)	49	19714.4	40958.7	106.21	0.00
40	age + time	age + log(time)	48	19722.1	40960.4	107.93	0.00
41	age + log(time)	age + log(time)	48	19720.4	40966.0	113.55	0.00
42	age x log(time)	age + log(time)	49	19726.8	40966.8	114.38	0.00
43	age x time^2	1	51	19735.7	40983.5	131.08	0.00
44	age x time	1	49	19739.0	40984.2	131.74	0.00
45	age + time^2	1	49	19743.0	40989.5	137.04	0.00
46	age + time	1	48	19742.8	40991.8	139.33	0.00
47	1	1	47	19745.7	40996.8	144.35	0.00
48	age + log(time)	1	48	19749.8	40998.6	146.15	0.00
49	age x log(time)	1	49	19760.0	40999.2	146.72	0.00

Figure S1. Hypotheses of invasive species population abundance (see Lockwood et al. 2013, Strayer et al. 2017) as a function of time since invasion. Such changes in abundance could have impacts on predator-prey dynamics and the emergence of sources and sinks. We note that as an invasion proceeds, one expects the increasing pattern to become either the density-dependence pattern, or to be the first phase of a boom-bust pattern.

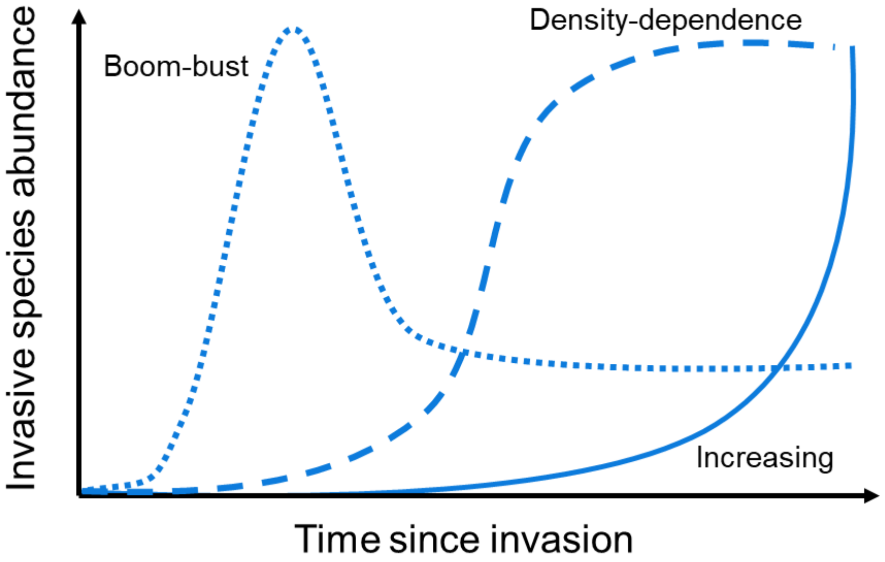

Figure S2. Snail kite movement probability between populations as a function of distance.

Predicted for an adult in the year 2013, at one, five, and ten years since invasion.

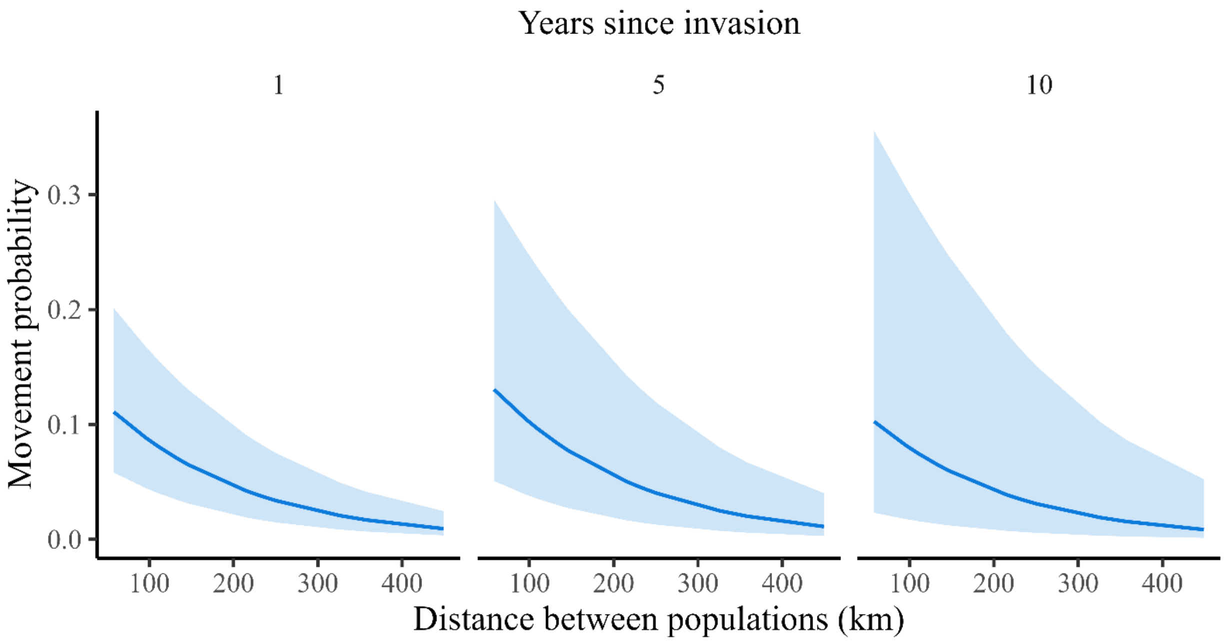

Figure S3. A snail kite nest in a flooded cattle field above *Pomacea maculata* egg clusters (the pink clumps at the base of the tree). Photo by Meghan A. Beatty.

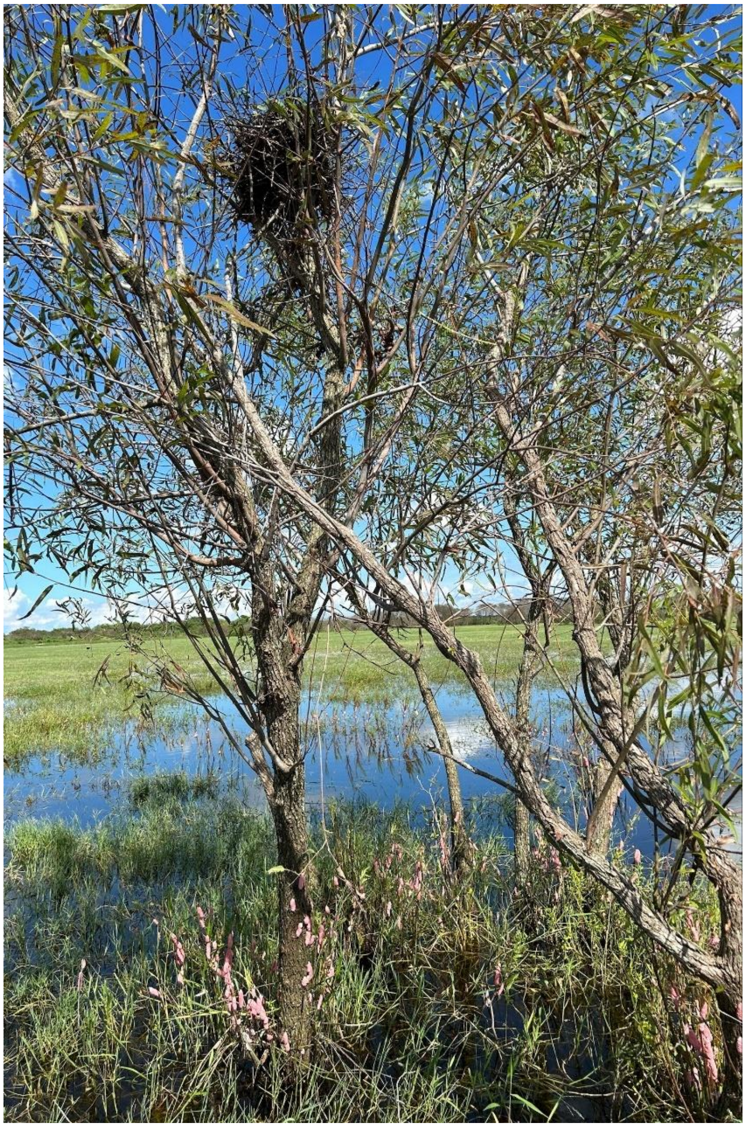

Figure S4. Estimates of survival probability across six snail kite populations as a function of years since *Pomacea maculata* invasion. There is no pre-invasion data on Paynes Prairie.

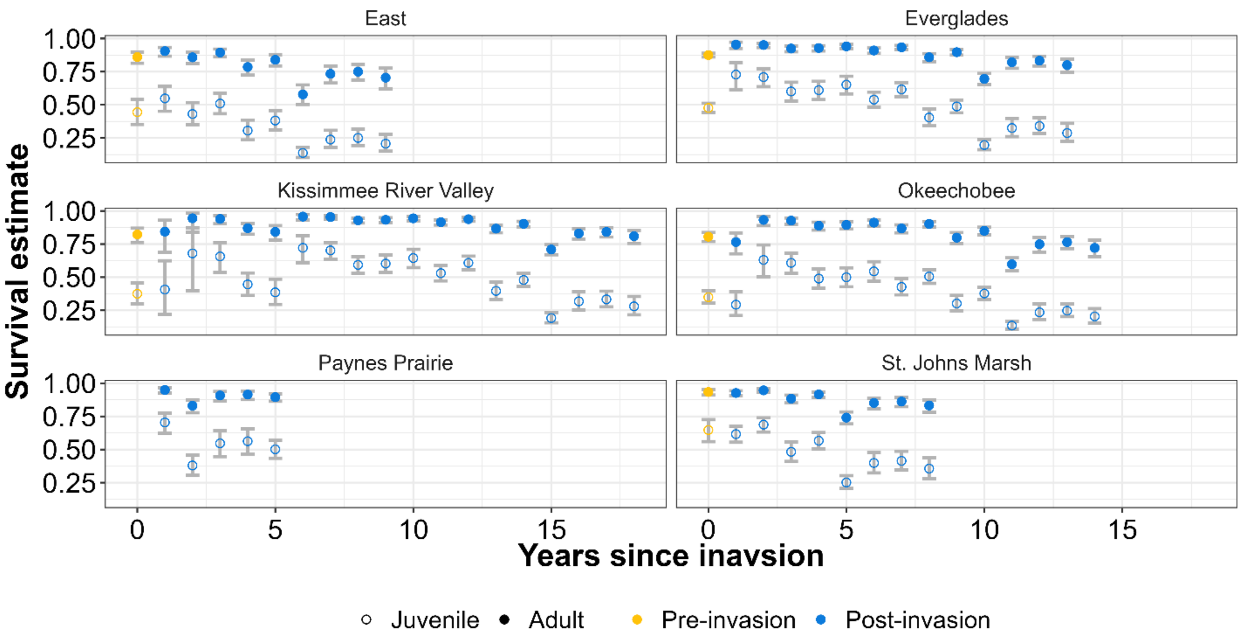

Figure S5. The proportion of years snail kite populations were sources or stable with $C_{rt} > 1$ after the invasion of *Pomacea maculata*. The number on top of the bar is the number of years since the population was invaded (i.e., the sample size). The dotted line represents the mean proportion of time all populations were sources.

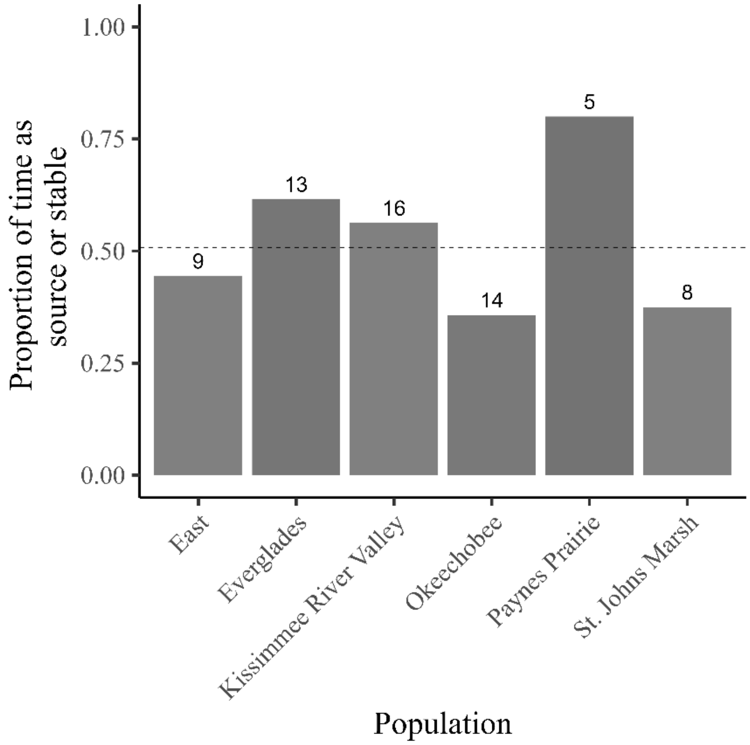

Figure S6. Source-sink estimates ($\boldsymbol{C_{rt}}$) for six snail kite populations as a function of local population count in the previous year. There was no significant relationship between $\boldsymbol{C_{rt}}$ and local population count.

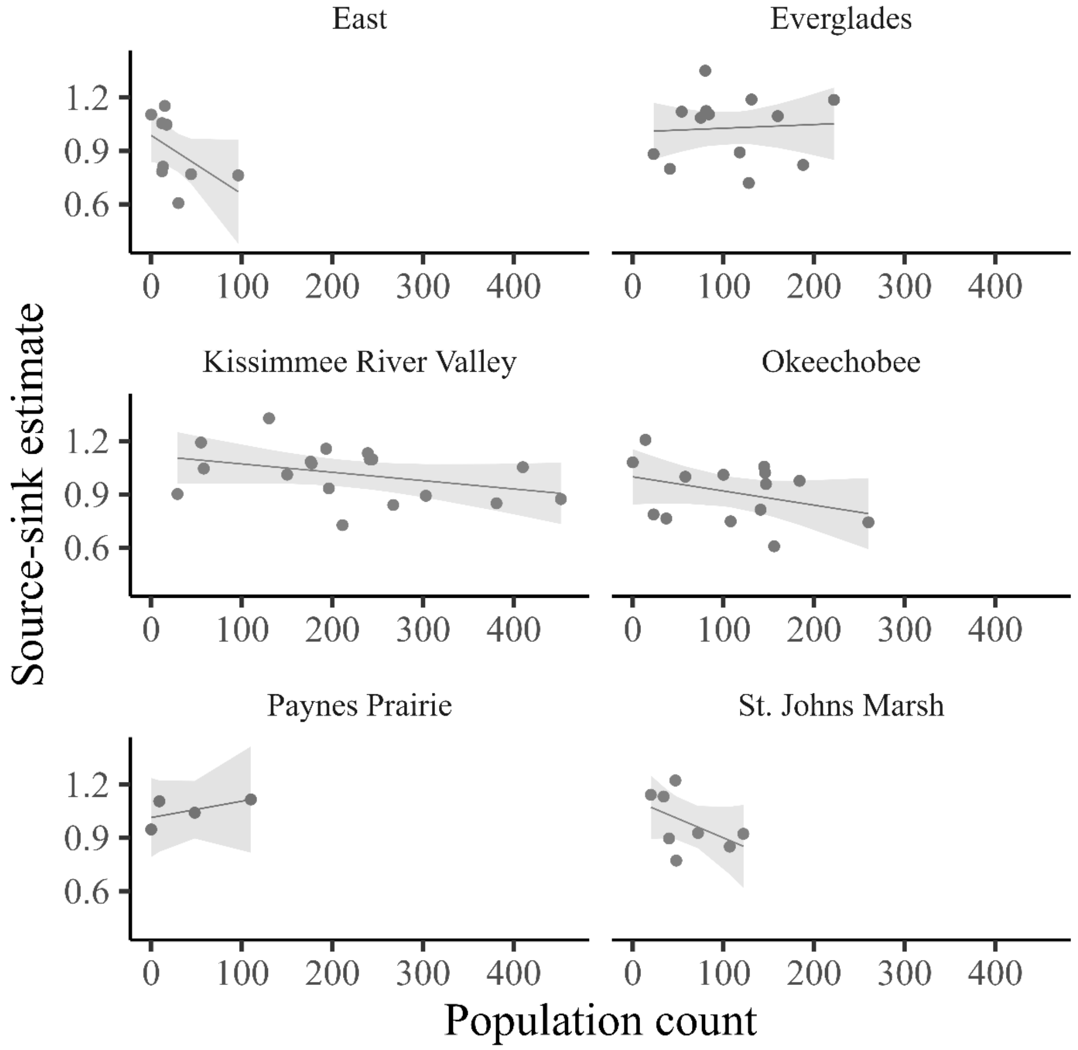

References for supplementary information

Beissinger, S. R. 1995. Modeling extinction in periodic environments: everglades water levels and snail kite population viability. Ecological Applications 5:618–631.

Bennetts, R. E., P. C. Darby, and L. B. Karunaratne. 2006. Foraging patch selection by snail kites in response to vegetation structure and prey abundance and availability. Waterbirds 29:88–94.

Benscoter, A. M., L. E. D'Acunto, S. M. Haider, R. J. Fletcher Jr., and S. S. Romañach. 2023. Nest-site selection model for endangered Everglade snail kites to inform ecosystem restoration. Ecosphere 14:e4362.

Cattau, C. E., R. J. Fletcher, B. E. Reichert, and W. M. Kitchens. 2016. Counteracting effects of a non-native prey on the demography of a native predator culminate in positive population growth. Ecological Applications 26:1952–1968.

Cattau, C. E., J. Martin, and W. M. Kitchens. 2010. Effects of an exotic prey species on a native specialist: Example of the snail kite. Biological Conservation 143:513–520.

Darby, P. C., D. J. Mellow, and M. L. Watford. 2007. Food-handling difficulties for snail kites capturing non-native apple snails. Florida Field Naturalist 35:79–85.

Fletcher Jr., R. J., M. A. Acevedo, B. E. Reichert, K. E. Pias, and W. M. Kitchens. 2011. Social network models predict movement and connectivity in ecological landscapes. Proceedings of the National Academy of Sciences of the United States of America 108:19282–19287.

Fletcher, R. J., C. Poli, B. Jeffery, M. Beatty, L. Elmquist, and M. Acevedo. 2024. Snail kite demography 2024 annual report on the 2023 breeding season. University of Florida, Gainesville, Florida, USA.

Fletcher, R. J., C. Poli, B. Jeffery, M. Beatty, and A. Gonzales. 2022. Snail kite demography 2022 annual report on the 2021 breeding season. University of Florida, Gainesville, Florida, USA.

Fletcher, R. J., C. Poli, E. Robertson, B. Jeffery, S. Dudek, and B. Reichert. 2017. Snail kite demography 2016 annual report. University of Florida, Gainesville, Florida, USA.

Fletcher Jr., R. J., A. Revell, B. E. Reichert, W. M. Kitchens, J. D. Dixon, and J. D. Austin. 2013. Network modularity reveals critical scales for connectivity in ecology and evolution. Nature Communications 4:1–7.

Fletcher, R. J., E. Robertson, S. Dudek, C. Poli, and B. Jeffery. 2019. Snail kite demography 2019 annual report on the 2018 breeding season. University of Florida, Gainesville, Florida, USA.

Fletcher Jr., R. J., E. P. Robertson, R. C. Wilcox, B. E. Reichert, J. D. Austin, and W. M. Kitchens. 2015. Affinity for natal environments by dispersers impacts reproduction and explains geographical structure of a highly mobile bird. Proceedings of the Royal Society B: Biological Sciences 282:1–7.

Fletcher, R. J., E. P. Robertson, C. Poli, S. Dudek, A. Gonzalez, and B. Jeffery. 2021. Conflicting nest survival thresholds across a wetland network alter management benchmarks for an endangered bird. Biological Conservation 253:108893.

Florida Fish and Wildlife Conservation Commission [FWC]. 2024. Annual report of activities conducted under the cooperative aquatic plant control program in florida public waters for fiscal year 2023 - 2024. Florida Fish and Wildlife Conservation Commission, Invasive Plant Management Section, Tallahassee, Florida, USA.

Glasheen, P. M., C. Calvo, M. Meerhoff, K. A. Hayes, and R. L. Burks. 2017. Survival, recovery, and reproduction of apple snails (Pomacea spp.) following exposure to drought conditions. Freshwater Science 36:316–324.

Gonzalez, A., and R. J. Fletcher Jr. 2025. Causes of nest failure vary with water depth, nesting substrate, and adverse weather in the endangered wetland-breeding Rostrhamus sociabilis plumbeus (Everglade Snail Kite). Ornithological Applications 127:1–12.

Kyle, C. H., A. L. Plantz, T. Shelton, and R. L. Burks. 2013. Count your eggs before they invade: identifying and quantifying egg clutches of two invasive apple snail species (Pomacea). PLOS ONE 8:e77736.

Laake, J. L. 2013. RMark: An R interface for analysis of capture-recapture data with MARK. Page 25. AFSC Processed Rep., Alaska Fish. Sci. Cent., NOAA, Natl. Mar. Fish. Serv., Seattle, WA.

Lockwood, J. L., M. F. Hoopes, and M. P. Marchetti. 2013. Invasion Ecology. John Wiley & Sons.

Mooij, W. M., R. E. Bennetts, W. M. Kitchens, and D. L. DeAngelis. 2002. Exploring the effect of drought extent and interval on the Florida snail kite: Interplay between spatial and temporal scales. Ecological Modelling 149:25–39.

Pias, K. E., R. J. Fletcher, and W. M. Kitchens. 2016. Assessing the value of novel habitats to snail kites through foraging behavior and nest survival. Journal of Fish and Wildlife Management 7:449–460.

Poli, C. L., K. D. Meyer, P. C. Darby, S. J. Dudek, G. Kent, and R. J. Fletcher Jr. 2024. Foray movements are common and vary with natal habitat for a highly mobile bird. Ecology and Evolution 14:e11096.

Poli, C., E. P. Robertson, J. Martin, A. N. Powell, and R. J. Fletcher. 2022. An invasive prey provides long-lasting silver spoon effects for an endangered predator. Proceedings of the Royal Society B: Biological Sciences 289:20220820.

Reichert, B. E., R. J. Fletcher, and W. M. Kitchens. 2021. The demographic contributions of connectivity versus local dynamics to population growth of an endangered bird. Journal of Animal Ecology 90:1–11.

Schwarz, C. J., J. F. Schweigert, and A. N. Arnason. 1993. Estimating migration rates using tag-recovery data. Biometrics 49:177–193.

Snyder, N. F. R., S. R. Beissinger, and R. E. Chandler. 1989. Reproduction and demography of the Florida Everglade (snail) kite. The Condor 91:300–316.

South Florida Water Management District [SFWMD]. 2023. Chapter 2A: South Florida Hydrology and Water Management. Pages 2A1-2A33 2023 South Florida Environmental Report – Volume I. South Florida Water Management District, West Palm Beach, Forida, USA.

Strayer, D. L., C. M. D'Antonio, F. Essl, M. S. Fowler, J. Geist, S. Hilt, I. Jarić, K. Jöhnk, C. G. Jones, X. Lambin, A. W. Latzka, J. Pergl, P. Pyšek, P. Robertson, M. von Schmalensee, R. A. Stefansson, J. Wright, and J. M. Jeschke. 2017. Boom-bust dynamics in biological invasions: towards an improved application of the concept. Ecology Letters 20:1337–1350.

White, G., and K. Burnham. 1999. Program MARK: Survival estimation from populations of marked animals. Bird Study 46 Supplement:120–138.
